# Expanded Spatiotemporal Concept of Cortical Visual–Vestibular Interaction in Humans: A fMRI Study on Visually Induced Motion Perception

**DOI:** 10.1002/brb3.71621

**Published:** 2026-07-24

**Authors:** Rainer Boegle, Franziska Reichl, Lena Fabritius, Maximilian Maywald, Oliver Pogarell, Thomas Brandt, Marianne Dieterich, Sandra Becker‐Bense

**Affiliations:** ^1^ German Center for Vertigo and Balance Disorders University Hospital, LMU Munich Munich Germany; ^2^ Graduate School of Systemic Neuroscience, LMU Munich Munich Germany; ^3^ Department of Neurology University Hospital, LMU Munich Munich Germany; ^4^ Department of Psychiatry and Psychotherapy University Hospital, LMU Munich Munich Germany; ^5^ Munich Cluster of Systems Neurology (SyNergy) Munich Germany

**Keywords:** coherent visual motion, fMRI, human, motion after effect, self‐motion perception, visual–vestibular interaction, visual–vestibular offset recalibration

## Abstract

Purpose: This high‐resolution fast fMRI study explores visual–vestibular network interactions in expanded spatial and temporal detail. Under natural conditions coherent visual motion always occurs during self‐motion. Technology applying coherent visual motion on stationary subjects creates visual–vestibular mismatch and allows investigation of system interplay in the MRI. In humans, the process minimizing the intersensory conflict was previously conceptualized as a categorical reciprocal upregulation of one (e.g., visual) and downregulation of the other system (e.g., vestibular), or vice versa.

Methods: Healthy participants (*n* = 22) were tested with a two‐phase paradigm using different coherent visual dot motion stimulation conditions (stimulation phase) and subsequent variable duration illusory self‐motion perception while the visual pattern was stationary, called motion aftereffect (MAE, post‐stimulation phase). A matched random dot stimulus, not inducing self‐motion perception, served as the control. Signal modulations related to subjective illusory self‐motion (MAE duration) were identified in a multivariate temporal model‐free way.

Finding: Visual areas showed uniform positive signal curves with motion stimulation unrelated to self‐motion perception (MAE duration), except for V5/MT. In contrast, vestibular areas exhibited fourfold signal patterns along the dimensions of transient versus sustained decreases and in relation to the stimulus phases and presence of correlation with MAE duration. Notably, beside V5/MT, further areas with intermediate signaling were identified.

Conclusion: Altogether, in contrast to the visual system, the vestibular network areas exhibited greatly differentiated processing instead of a categorical uniform system “on‐off” mode. These novel findings expand our comprehension of the continuous interaction between the visual and vestibular systems, especially with respect to the various dynamical activity changes in vestibular areas, potentially indicating subspecialization of regions that might be essential even for humans moving in the real world.

## Graphical abstract

The vestibular system plays a key role in self‐motion processing. Reciprocal visual–vestibular interaction is differentiated, not an on‐off mode. The human vestibular system shows subspecialization for motion stimulus properties.

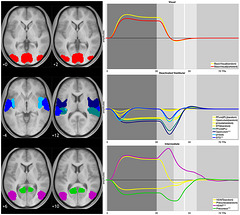

## 1 Introduction

For maintaining perceptual stability during movement, as well as adequate posture and balance control, a multisensory process mainly recruiting vestibular and visual inputs is essential. The visual and vestibular systems must simultaneously negotiate the relationship of motion estimates between the two sensory inputs into a coherent whole movement estimate. Under natural conditions, coherent visual motion (e.g., optic flow) always occurs during self‑motion, and the resulting motion estimate from the visual system is always in the opposite direction to the motion estimate from the vestibular system (DeAngelis and Angelaki 2012). However, modern displays allow viewing coherent visual motion while stationary, creating a visual‑vestibular mismatch. Prolonged exposure to this mismatch (the stimulation phase) leads to a motion aftereffect (MAE) when a stationary pattern is presented afterward (post‐stimulation phase), that is, the stationary pattern seems to move in the opposite direction of the previously presented coherent visual motion, gradually slowing down and finally ceasing (Wohlgemuth 1911; Harris et al. 1981). It was shown that MAE, from large‐field patterns, elicits a perception of global motion, that is, a kind of vection, caused by involvement of the vestibular system, hence visual–vestibular interaction (Harris et al. 1981; Hiris and Blake 1992). Since during stimulation there is visual motion activity, and post‐stimulation there is no longer visual motion but illusory (vestibular) motion, the MAE stimulus can be used in the MRI to study contribution of both systems, visual and vestibular, in one paradigm. By grading the visual motion input and MAE duration, it is further possible to evaluate a kind of dose–response relationship by correlation with the BOLD signal.

Two lines of research have addressed visual–vestibular interaction in humans so far: Earlier neuroimaging studies (PET and fMRI studies) examining only the period of coherent motion stimulation found a reciprocal pattern: activation of visual parieto‑occipital areas (including V5/MT) and concurrent deactivation of the vestibular cortical network, with the posterior insula/operculum as its core region (Brandt et al. 1998, 2002). This reciprocal activation‑deactivation was interpreted as a categorical “on‑off” mechanism for resolving intersensory conflict at cortical level by shifting sensory weighting from one modality to the other (Brandt et al. 1998; Dieterich and Brandt 2020). This view was enforced by demonstration of a reversed pattern of response (visual deactivation and vestibular activation) during vestibular stimulation (for review, see Dieterich and Brandt 2020, 2024). Importantly, due to methodological limitations (limited temporal resolution in PET and block‐average fMRI), these neuroimaging studies on visual stimulation were not able to evaluate the signal pattern over the whole paradigm, especially not in the post‑stimulation phase of the MAE.

Behavioral studies, in contrast, using MAE as a window into the processing of motion estimates from coherent visual motion and applying vestibular intervention stimuli, have demonstrated that the aftereffect cannot be explained simply by reciprocal suppression. For example, moving during the stimulation phase leads to modulations of the MAE duration, and furthermore, the fact that MAE can be “stored,” that is, decay of MAE only happens once a stationary pattern is presented (Bai et al. 2019, 2020; Harris et al. 1981; Mather et al. 2008; Van De Grind et al. 2004). Therefore, it was proposed that during prolonged coherent motion stimulation, the brain adjusts an inter‐system offset (Bai et al. 2019, 2020,; Cuturi 2022; Cuturi and Macneilage 2014; Harris et al. 1981; Hiris and Blake 1992). While the visual system signals a consistent non‑zero velocity (e.g., 10°/s) during visual stimulation, the vestibular system signals zero motion (stationary). It was proposed that the brain gradually shifts an internal offset between the systems to minimize this discrepancy by adjusting the visual–vestibular inter‐system offset to −10°/s (Bai et al. 2019, 2020; Harris et al. 1981). When the visual stimulation stops afterward and a static pattern appears, this newly established offset becomes perceptible as illusory motion (MAE), and the inter‐system offset must be reset back to zero. This resetting process was postulated to reflect the “slowing down” of MAE percept and the fact that MAE can be “stored,” that is, reset of the inter‐system offset only happens once both systems signal stationarity upon showing a static pattern (Bai et al. 2019, 2020; Harris et al. 1981; Hiris and Blake 1992; Jaekl et al. 2005; Seno et al. 2015). Taken together, this necessitates an active process of continuous integration during both stimulus phases.

Here, we were able to combine both approaches. First, we used high‑resolution fMRI (Uǧurbil et al. 2013) to record BOLD responses throughout the entire trial (stimulation and post‑stimulation), and we applied different coherent visual motion conditions leading to different length of MAE duration, hence a dose–response relationship. Second, model‐free analysis was used, splitting whole‐brain activity into networks (independent component [IC] analysis) with one signal time course per network (Beckmann and Smith 2004) and applying correlation analysis between signal time course and motion percept via partial least squares (PLS) correlation (Castelo‐Branco et al. 2009; McIntosh and Lobaugh 2004).

Our hypotheses were:

1. The cortical reciprocal interaction concept might be widened with respect to involvement of the meanwhile well‐known multiple vestibular network areas, beside the vestibular core region in the posterior insula/operculum region, and possibly further areas related to visual–vestibular interaction.
2. The temporal properties within the different areas and networks will show signal behavior beyond a simple categorical “on‐off” mode of inter‐systems interaction, that is, not only mismatch‐related reciprocal responses but continuously changing signal patterns related to the ongoing adjustment of the inter‐system offset.

It was expected that this higher temporal and spatial resolution would reveal a deeper understanding of when, how, and to what extent various areas are related to the intersensory system interaction at cortical level providing important puzzle pieces in the overall understanding of how moving might be achieved in the real world.

## 2 Materials and Methods

### 2.1 Healthy Participants

Twenty‐two healthy participants (12 females, mean age 32 ± 12 years) were recruited (by word of mouth and notice boards in the clinic area) between February and May of 2023. All were examined clinically, including vestibular and balance testing, to rule out subclinical neurological and vestibular deficits. All 22 participants were right‐handed (Oldfield 1971; Salmaso and Longoni 1985).

The study was conducted according to regulations of the Helsinki Declaration (revised 2024) and was approved by the local Ethics Committee of the Ludwig‐Maximilians‐Universität München, Germany (23‐0660). All participants gave their informed written consent.

### 2.2 MRI Data Acquisition

Imaging data acquisition was performed on a 3T MR scanner (type “Skyra” by Siemens Erlangen located at the university hospital of the LMU in Großhadern, Munich, Germany) with a 64‐channel head and neck coil. Functional MRI data were recorded using a multiband echo‐planar imaging sequence (1445 EPI volumes, 64 × 64 × 54 isotropic voxels, voxel size 2.5 mm, TR 700 ms, multiband factor 6, TE 33 ms) in axial orientation (Smith et al. 2013; Uǧurbil et al. 2013). Fifteen initial dummy scans were discarded to account for T1 saturation effects of the MRI signal. Structural imaging consisted of a high‐resolution T1‐weighted scan with isotropic voxel size of 0.75 mm, using an MPRAGE sequence (Brant‐Zawadzki et al. 1992).

### 2.3 Stimulation Paradigm and Experimental Procedure

**FIGURE 1:**
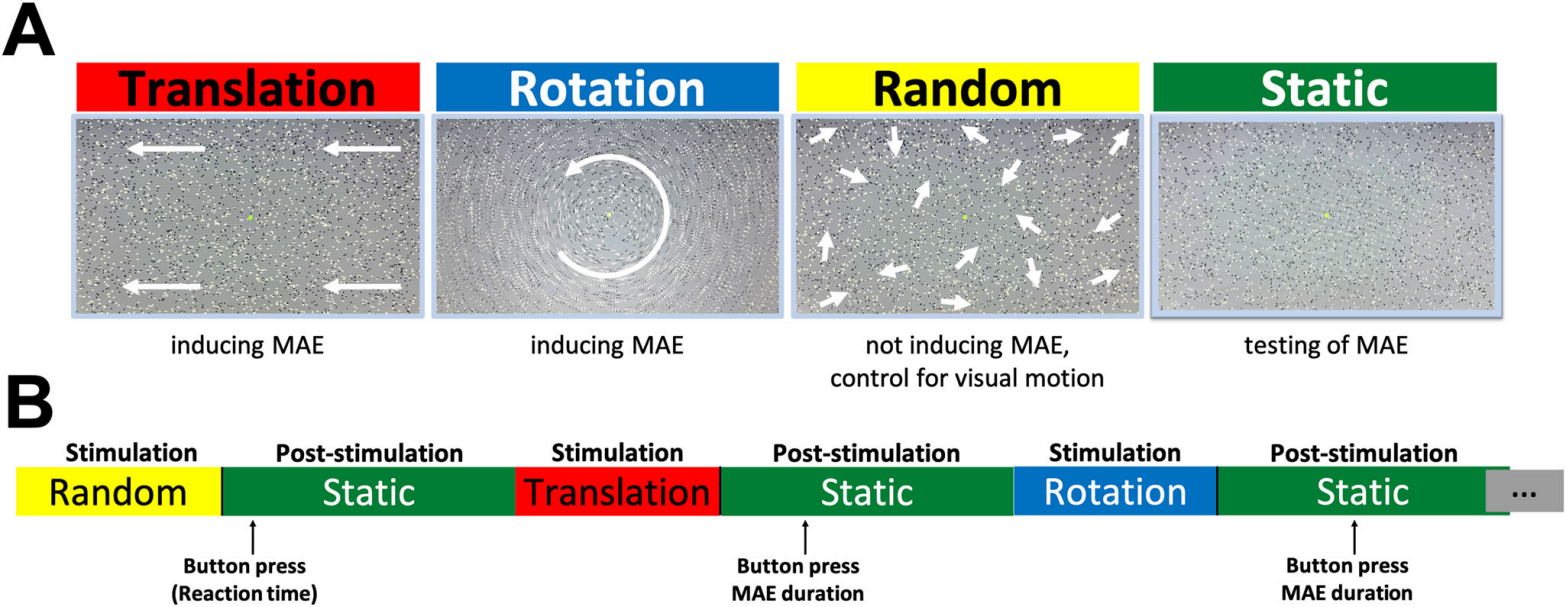
(A) Visual dot motion stimulation conditions used in the experiment (black and white dots on a gray background, green fixation dot at the center). The direction of motion is indicated by white arrows. (B) Excerpts of a typical MRI timeline including the different visual motion conditions (translation, rotation, and random) applied in pseudorandomized order, each followed by the static dot pattern. Exemplary button press times indicating the end of perceived MAE (self‐motion) are given.

We used a two‐phase visual motion paradigm, the parts of which we refer to as the “stimulation phase,” during which moving dots are seen, and the “post‐stimulation” phase after a sudden stimulus stop, during which static dots are seen. For stimulation, three conditions of the same moving dot pattern were applied: (a) coherent right‐to‐left translational dot motion stimulation, (b) coherent counterclockwise rotational dot stimulation, and (c) incoherent random dot motion stimulation (of the same velocity) (Figure 1). During the post‐stimulation phases, the same static dot pattern was always presented. In the translation and rotation conditions, illusory self‐motion of varying degrees between subjects and trails is expected, but not following the random dot motion (control for visual input per se).

The visual dot pattern consisted of 800 black and white dots (diameter 0.1°, placed randomly) on a gray background (Figure 1). All dots were animated to move with a velocity of 6°/s. Additionally, a green fixation dot was present at the center of the screen during all stimulation and post‐stimulation periods to suppress eye movements. Participants were instructed to fixate on the central green dot, and to not follow the moving dots with their eyes, but to attentively capture the motion percept during stimulation as well as during the post‐stimulation phase. Stimuli were generated with the PsychToolbox (V3.0.18 http://psychtoolbox.org/) using MATLAB R2017 on a Dell Laptop situated in the control room and connected to the screen in the MRI room.

Presentation was done on a display screen (NNL, NordicNeuroLab AS, Bergen, Norway, https://www.nordicneurolab.com/) with resolution 1920 × 1080 pixels and diagonal 32″, situated at the head end of the MRI magnet bore and observed by the participants through a semi‐transparent mirror fixed to the head coil. All stimulation phases lasted for 30 TRs, followed by a post‐stimulation phase for 45 TRs. Since the MAE cannot last longer than the stimulus length (and is usually much shorter), there was enough time in the post‐stimulation phase for the BOLD response to return to baseline. Each of the three motion conditions had six repetitions that were presented in pseudorandomized order over the run. A constraint that each condition would appear twice in the first, second (middle), and third (last) segments of the run was added in order to ensure that each condition appears roughly equally distributed over the run.

The participants indicated the end of their MAE perception by a right‐handed button press after each trail of the translation and rotation condition. For the random condition (not inducing MAE), they indicated the moment when they felt confident that motion stimulation had stopped, that is, reflecting the reaction time. Button presses were recorded via fiber optic transmission input coming from the MRI room to the control room, using CurrentDesigns device fORP 904 bimanual (https://www.curdes.com). The MAE duration was calculated from the end of the stimulation phase till the button press and averaged over all trials per condition and participant. MAE durations between conditions were compared with a paired signed‐rank test. After the experiment, participants were debriefed about their experience during the experiment. Furthermore, the participant's right eye was recorded with an infrared video camera (NNL, NordicNeuroLab AS), and videos were saved using View Point Eye Tracker software (Arrington Research, www.arringtonresearch.com), which included synchronization markers from the MRI (aligning video frames with experiment conditions based on MRI volume) for control of proper task performance, for example, alertness/attention, maintaining fixation, and not falling asleep.

In order to allow statistical modeling of all time points of the stimulation and post‐stimulation phases without producing an overdetermined model, an extra “blank trail” condition was added. This “blank trail” condition randomly appeared over the whole run with nine repetitions in total, that is, 50% probability after a post‐stimulation phase. A random “blank trail” consisted of a period of 8 TRs without presentation of any dots and only the gray background screen visible. This resulted in 72 TRs of “blank trail” in each run. The additional benefit of these “blank trails” was to allow the participants to relax briefly, prevent eye strain from constant fixation, and remind them to keep attending to the task with the reappearance of the green fixation dot. All in all, this resulted in 1445 TRs, with 75 TRs for each of the six repetitions per condition (30 TRs stimulation and 45 TRs post‐stimulation phase) for three conditions, and additionally 15 TRs for dummy scans at the beginning of each run to allow for signal saturation, 72 TRs “blank trails,” and adding a final 8 TRs trailing the last post‐stimulation period.

Each stimulus onset time, offset time, button press time, and occurrence of MRI scanner volume triggers was recorded in a log file for extraction of event timing for later analysis. The latency for event recording was estimated (PsychToolbox) to be roughly 10–20 ms and thus far better than necessary compared to an fMRI repetition time of TR 700 ms and MAE durations of roughly 5–17 s.

### 2.4 MRI Data Analysis

We used SPM12 software and Linux workstations for imaging data processing (SPM v7771, http://www.fil.ion.ucl.ac.uk/spm/software/spm12/, MATLAB R2022b, https://www.mathworks.com/). All MRI image volumes were corrected for head motion by realignment to the mean image of the run. Every participant's structural scan was coregistered to the mean image of the realigned EPI images, followed by segmentation of the structural image. The parameters obtained were then applied (via flow fields) to warp all functional and structural images to the Montreal Neurological Institute standard space with a resolution of 2 mm × 2 mm × 2 mm. Functional data were smoothed using a Gaussian kernel of 5 mm FWHM and filtered temporally with a high‐pass filter of 128 s before statistical analysis.

The preprocessed fMRI data were decomposed using group independent component analysis (gICA) implemented in FSL MELODIC (version 3.15, https://fsl.fmrib.ox.ac.uk/), that is, all fMRI data were concatenated temporally and reduced in dimension via principal component analysis, whitened, and the resulting components unmixed using fastICA negentropy estimation using the default exponential (pow3) nonlinearity and parameter settings of MELODIC (Beckmann and Smith 2004). The dimensionality of the gICA was set to 150 (retaining 79.54% of total variance), and higher number of dimensions only marginally increased explained variance, for example, 200 dimensions explaining 81% of total variance, only about 2% more variance. Hence, by the generally accepted “knee method,” we settled on 150 dimensions as sufficient. The thresholding of spatial maps after decomposition was done via multivariate mixture model thresholding (one Gaussian and two gamma distributions). Thresholding used the default threshold of *p* > 0.5, that is, declaring a voxel activated if it belongs to one of the gamma distributions rather than the Gaussian distribution.

Relevant ICs were selected for further analysis based on the following criteria:

1. If they were cortical regions, not associated with noise regions (blood vessel‐associated pulsation, white matter fluctuations, cerebrospinal fluid fluctuations, or movement‐related artifacts).
2. If they showed a response during the stimulation phase and/or during the post‐stimulation phase.
3. If they showed a response modulation during the MAE period in relation to the individual MAE duration, indicating importance. See event‐related averaging and PLS analysis below.

Regions in the selected components were identified using the Juelich Histological and Harvard‐Oxford Structural atlases as included in the SPM anatomy toolbox (version 3 [Eickhoff et al. 2005]), and components were labeled according to the most prominent region (e.g., V1, V5/MT, etc.) listed in the atlas or the overall pattern of regions (default mode network [DMN], posterior insula, etc.).

### 2.5 Event‐Related Averaging of IC Time Courses

Each IC has a representative time course (contained in the mixing matrix) indicating the response of all areas belonging to the IC for each participant. In short, BOLD activity data for each trail per condition, participant, and component were extracted and averaged over trails to represent the response per condition, participant, and component. These averages were then used in the PLS correlation analysis, described below, together with the average MAE durations per condition and participant. The averages per condition and participant were then further averaged over participants to create a representative time course per condition for display in the figures.

The detailed steps were: Signal change time courses were extracted from the overall time course of each IC and separated into “snippets” starting 10 TRs before the onset of the stimulation until the end of the respective post‐stimulation phase, that is, in total 85 TRs (10 TRs or 7 s ahead, 30 TRs or 21 s stimulation, and 45 TRs or 31.5 s post‐stimulation phase). This was done for each trail of each condition per participant, hence an event‐related (the event was the onset of the stimulation phase) extraction of signal change. Although temporal filtering was used in the preprocessing of fMRI data before using gICA, it is possible that trends and changes of baseline between trails remain in the IC time courses. Therefore, the value of the extracted time course snippet at the onset time point of the stimulation phase was subtracted from each time point of the snippet to make all changes relative to the onset of stimulation, and linear detrending was added for each “snippet.” All trails per condition and participant were then averaged. These representative ERAs per condition and participant were later used in the PLS correlation analysis described below as the mean response per condition and participant naturally related to the mean MAE duration per condition and participant. The ERAs per condition and participant were then further averaged over all participants for presentation of the average group response.

### 2.6 PLS Analysis of Relationship Between MAE Duration and ERAs

The relationship of MAE duration to the shape of ERA time courses was analyzed using PLS correlation (McIntosh and Lobaugh 2004), which we have used previously (Cyran et al. 2016). In short, instead of separately correlating each time point in the stimulation and post‐stimulation phases for each participant with the MAE durations of each participant, PLS correlation also correlates all time points weighted by the respective participant's MAE duration after z‐scoring both over the group. This means that the signal relationship over time is incorporated into the analysis (i.e., how earlier time point and MAE duration products are correlated to later time point and MAE duration products), and the result is the maximally accumulated possible correlation value over all time points for the given MAE durations. To assess significance, the MAE durations are randomized over participants and the process repeated, hence permutation testing, that is, asking the question if a similar or larger correlation value could be achieved if MAE durations were randomly assigned instead of proper assignment to the respective participant time course.

Expressed in more detail, PLS correlation is based on the singular value decomposition of a correlation matrix and reveals the multivariate relationship between a variable of interest (here, the MAE durations) and a multivariate response matrix (here, all the time courses of all participants’ ERAs). PLS finds the linear combination of the columns of the response matrix, which will produce maximum covariance with the variable of interest. Hence, PLS correlation between MAE durations and all ERA time courses produces one correlation coefficient, expressing the strength of relation between MAE duration and ERA time courses in a model‐free way. Statistical significance is assessed with permutation testing, that is, the MAE durations are permuted such that the assignment of MAE duration with the participant's ERA time course is lost and PLS correlation is repeated. If the correlation coefficient produced is equal or larger than the original one (unpermuted MAE duration), this is counted as a failure of the permutation test, because a random assignment produced a correlation coefficient of equal or greater magnitude. This process is repeated a large number of times (here, 10,000 permutations), and the statistical significance is determined as the number of failures divided by the total number of permutations. PLS correlation permutation testing was done for the complete ERA time courses, as well as the stimulation and the post‐stimulation separately. This was done for each component separately, and multiple comparison correction was applied via false discovery rate over all 150 components with *q* = 0.001 (Benjamini and Hochberg 1995).

In all figures, we marked the permutation test results as follows: If the number of failures was, for example, 1 in 10,000 (*p* = 0.0001) or less, it was marked as highly significant (***), 5 in 10,000 (*p* = 0.0005) or less, as significant (**), and 10 in 10,000 (*p* = 0.001) or less, as weakly significant (*). Any number of failures above 10 in 10,000 (*p* > 0.001) was considered as not significant (n.s.).

With this temporal model‑free approach, we were able to account for the entire temporal response, avoid simple block‑averaging limitations, and reveal which brain network dynamics correlate with individual differences in illusory motion strength (MAE duration).

## 3 Results

### 3.1 Behavioral Effects During the Stimulation and Post‐Stimulation Phase

**FIGURE 2:**
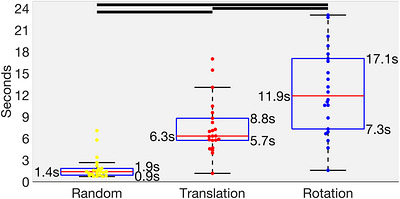
Behavioral data of perceived self‐motion. Box plots of MAE durations per condition indicated by button press. The rotation condition (blue) and the translation condition (red) reliably induced MAE (illusory self‐motion perception), and the random condition (yellow, reflecting reaction time) never did. The mean MAE durations differed significantly between the conditions (rotation > translation > random). The thick black lines at the top indicate significant paired signed‐rank test (*p* < 0.001).

As intended, all participants reported clear illusory global motion perception (MAE) during the post‐stimulation phase of all translational and rotational stimulations in the opposite direction, but never after the random stimulation. The durations until button press differed significantly between all stimulation conditions in the paired signed‐rank test (*p* < 0.001). They were longer for the rotation condition than for the translation condition: 11.9 s (interquartile range 7.3–17 s) versus 6.3 s (interquartile range 5.7–8.8 s), respectively (Figure 2). The random condition, reflecting reaction time, had the shortest time till button press with 1.4 s (interquartile range from 0.92 to 1.9 s).

### 3.2 Motor and Salience‐Attention‐Executive Network Components

**FIGURE 3:**
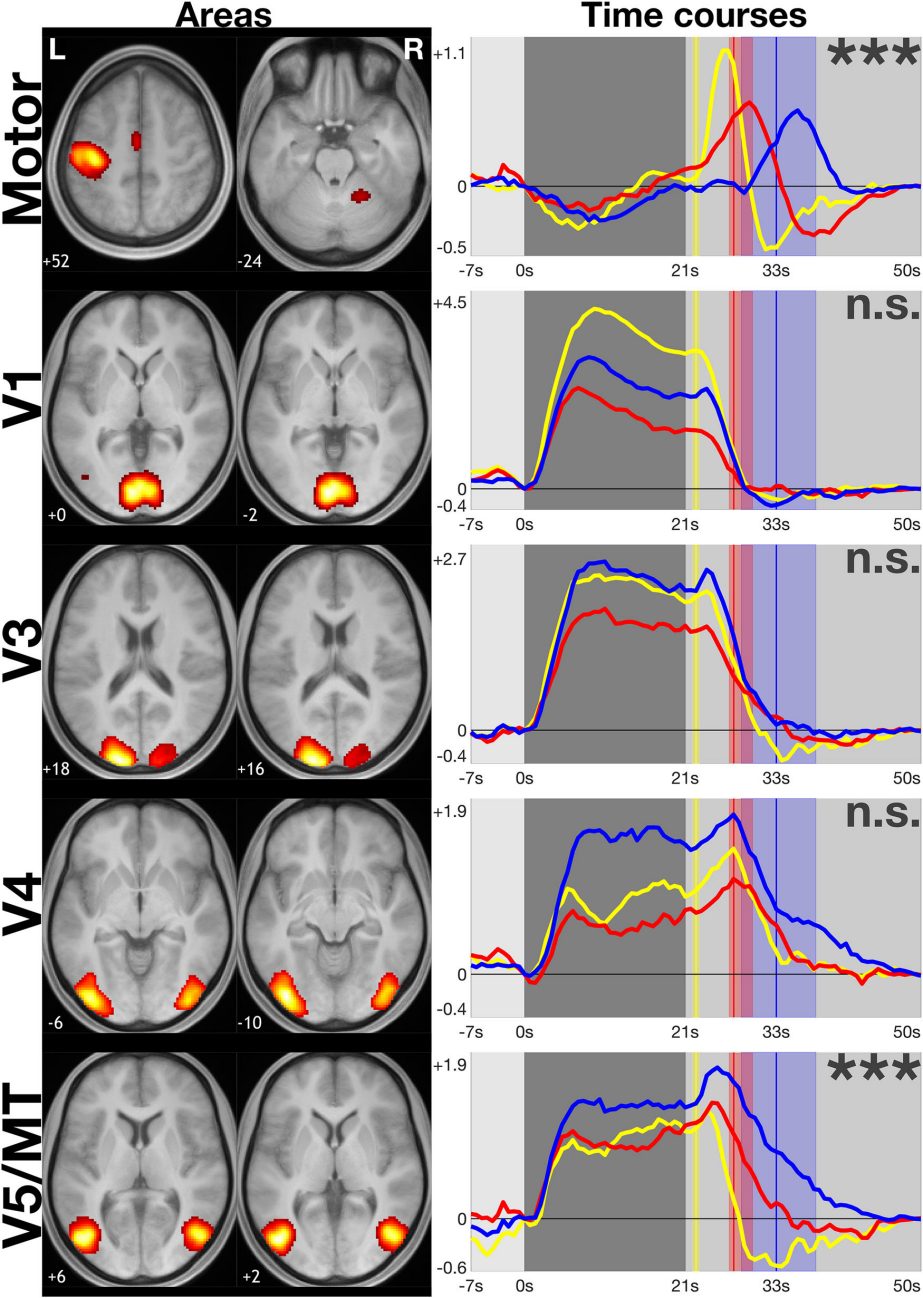
Temporal and spatial properties of motor and visual components. The right‐handed button press led to a post‐stimulation response in the left (pre‐)motor cortex, the SMA, and right cerebellum, first after random, second after translational, and last after rotational stimulation. For the visual components V1, V3 and V4, the responses for all three conditions rapidly return to baseline after stimulus stop in a very similar timely manner independent of MAE duration, also reflected by their PLS correlation not being significant. In contrast, the V5/MT component shows clear distinctions in returning to baseline between conditions with random returning first, translation second, and rotation last, which is also reflected in the highly significant PLS correlation. Left column: overlays of activated areas; right column: responses relative to onset of visual motion stimulation. Time course illustration: Response curves color‐coded in blue for rotation, in red for translation, and yellow for random. Stimulation phase indicated by dark gray (0 = Onset to 30TRs), and the post‐stimulation phase by light gray background (30–70 TRs). Translucent vertical color regions illustrate when 25% (left edge) or 75% (right edge) of the group perceived the end of MAE during a condition, respectively. The central vertical line indicates when 50% of participants perceived the end of MAE. *** highly significant PLS correlation with MAE duration (*p* < 0.0001); n.s. = not significant.

The right‐handed button press led to expected responses primarily in the left (pre‐)motor cortex, as well as in the supplementary motor area and right cerebellum (VI/V) depending on the button press time, first after random, second after translational, and last after rotational stimulation (Figure 3). With random dot stimulation, they were most focused over time as all participants pressed the button roughly at the same time. For the translation and rotation conditions, the peaks were a bit more spread out (Figure 3). These different response curves indicated the time frames in which a modulation of components due to MAE was expected.

**FIGURE 4:**
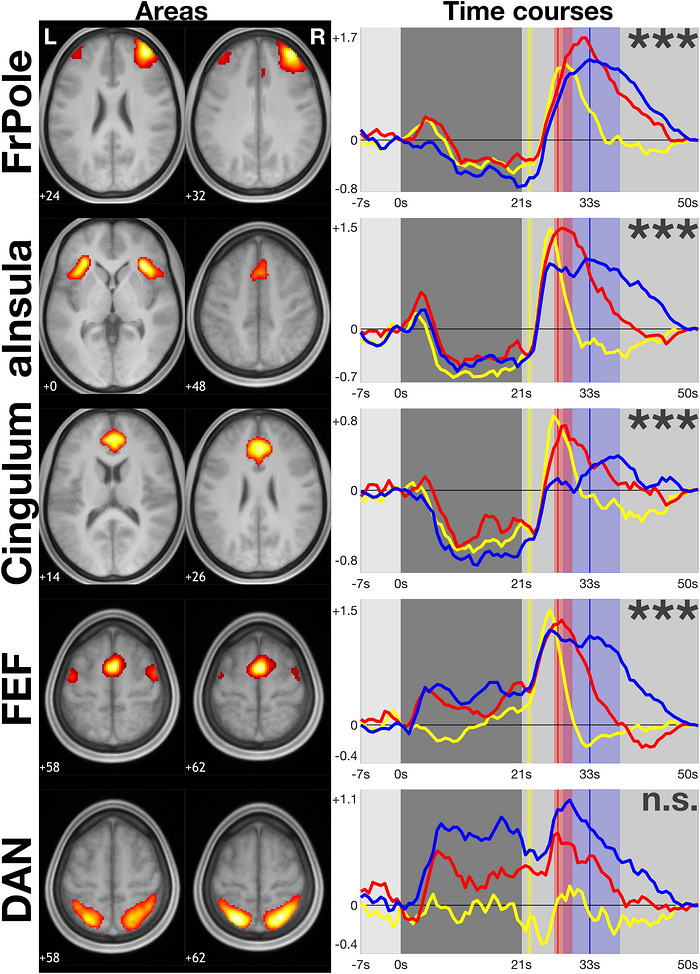
Temporal and spatial properties of salience‐attention‐executive components. Frontal pole (FrPole), anterior insula (aInsula), and anterior cingulum showed a negative response during the stimulation phase and a positive response during the post‐stimulation phase. The responses returned to baseline in a differentiated timely manner in relation to MAE durations: first random condition, second translation, and last rotation. The frontal eye field (FEF) showed a slight positive response during the stimulation phase for translation and rotation, but almost none for random. The return to baseline in FEF analogously to the aforementioned areas varied between the conditions in relation to MAE duration. The dorsal attention network (DAN) showed a positive response for translation and rotation during the stimulation phase, slightly increasing at the start of the post‐stimulation phase before returning to baseline in a uniform timely manner. The random condition did not elicit a clear response in DAN. Left column: overlays of activated areas; right column: responses relative to onset of visual motion stimulation. Time course illustration: Response curves color‐coded in blue for rotation, red for translation, and yellow for random. Stimulation phase indicated by dark gray (0 = Onset to 30TRs), and the post‐stimulation phase by light gray background (30–70 TRs). Translucent vertical color regions illustrate when 25% (left edge) and 75% (right edge) of the group perceived the end of MAE during a condition, respectively. The central vertical line indicates when 50% of participants perceived the end of MAE. *** highly significant PLS correlation with MAE duration (*p* < 0.0001); n.s. = not significant.

Since the study design demands high attention to keep fixating, monitor the overall visual scene, notice the end of MAE, and plan the button press, there were responses in various parts of the salience‐attention‐executive networks: the frontal pole, anterior insula, anterior cingulum, frontal eye field (FEF), and dorsal attention network (DAN) (Figure 4; see also Figure S1). The first three of these areas all showed a negative response during the stimulation and a positive response during the post‐stimulation phase. The responses in FEF were slightly positive during the stimulation phase and increased further in the post‐stimulation phase. In accordance with the varying MAE durations for random, translation, and rotation, these responses successively returned to baseline and showed a significant PLS correlation with MAE duration (Figure 4, Table 1). In contrast, the DAN showed responses only during coherent‐motion stimulation (translation and rotation), but not during the incoherent random condition, and did not show a significant PLS correlation with the MAE durations (see Figure S1 with extra components of similar response type).

**TABLE 1:** Results of the partial least squares (PLS) correlation analysis between MAE durations and IC ERA time courses of each participant. The components in bold were those for which a significant relationship between MAE durations and ERA time course were found. The correlation coefficients and *p*‐values are listed, as well as the details of the peak MNI coordinates and cluster sizes of the relevant areas.

Fig	IC	Labels	Post‐stimulation phase	All timepoints	Stimulation phase				
						Cluster1 nVoxels, XYZmmMNI(z‐value)	Cluster2 nVoxels, XYZmmMNI(z‐value)	Cluster3 nVoxels, XYZmmMNI(z‐value)	Atlas labels
3	5	V1	corr = 0.1991	corr = 0.2673	corr = 0.2785	2540 Voxels			hOc1[V1],hOc2[V2]—Lingual Gyrus
						[−6, −86, −2](39.3)			
			pPerm = 0.5221	pPerm = 0.1831	pPerm = 0.0358	[6, −84, 0](32.4)			
3	84	V3	corr = 0.3299	corr = 0.2917	corr = 0.144	1832 Voxels	510 Voxels		hOc3d[V3d],hOc4d[V3A],hOc2[V2]—Occipital Pole
						[−18, −96, +18](26.9)	[14, −96, +20](12.3)		
			pPerm = 0.035	pPerm = 0.1804	pPerm = 0.5892				
3	57	V4	corr = 0.3194	corr = 0.3272	corr = 0.2972	2065 Voxels	1067 Voxels		hOc4v[V4v], FG1, FG2, hOc41a, hOc41p—Lateral Occipital Cortex inferior division
						[−38, −80, −14](21.9)	[+40, −84, −8](16.1)		
			pPerm = 0.0779	pPerm = 0.1571	pPerm = 0.0475	[−42, −74, −16](20.3)			
3	85	**V5/MT**	**corr = 0.6664**	**corr = 0.6224**	corr = 0.2886	1410 Voxels	1268 Voxels		hOc5[V5/MT], hOc41a—Lateral Occipital Cortex inferior division
						[−46, −74, +2](27.9)	[+48, −70, +4](24.7)		
			**pPerm = 0**	**pPerm = 0**	pPerm = 0.0999		[+44, −68, +0](24.2)		
3	2	**Motor**	**corr = 0.5813**	**corr = 0.5768**	corr = 0.1346	3133 Voxels	417 Voxels	335 Voxels	Area 4a—Precentral Gyrus; Cerebellum Right VI/V; Area 6mc/SMA—Juxtapositional Lobule Cortex, Cingulate Cortex posterior division
						[−38, −20, +60](57.9)	[+20, −52, −22](16.3)	[−2, −8, +52](18)	
			**pPerm = 0**	**pPerm = 0**	pPerm = 0.721			[−6, −22, +46](10.5)	
4	11	**FrPole**	**corr = 0.6477**	**corr = 0.693**	corr = 0.3802	2486 Voxels	264 Voxels	261 Voxels	Frontal Pole; Middle Frontal Gyrus
						[+36, +50, +28](24.6)	[+20, +14, +66](10.9)	[−38, +44, +30](9.66)	Superior Frontal Gyrus
			**pPerm = 0**	**pPerm = 0**	pPerm = 0.0098			[−32, +46, +36](7.92)	
4	35	**aInsula**	**corr = 0.6713**	**corr = 0.6996**	corr = 0.1467	1294 Voxels	1063 Voxels	900 Voxels	Area Id7, Area OP9 ‐ Frontal Operculum Cortex; Insula Cortex; Area 6mr—Paracingulate Gyrus; Superior Frontal Gyrus; Cingulate Gyrus anterior division; Area p32, Area p24c
						[+34, 26, +2](19.6)	[−30, +26, +4](18.5)	[+6, +20, +44](11.5)	
			**pPerm = 0**	**pPerm = 0**	pPerm = 0.8813	[+38, +22, −6](17.8)	[−30, +26, −6](16.2)	[+2, +18, +48](11.2)	
								[−4, +14, +50](10.1)	
								[+4, +32, +40](8.54)	
								[+8, +36, +30](8.13)	
								[−4, +30, +34](8.1)	
								[+10, +28, +32](7.55)	
4	49	**Cingulum**	**corr = 0.6407**	**corr = 0.5998**	corr = 0.1617	2439 Voxels			Area p24ab, Area p32, Area p24c—Cingulate Gyrus anterior division; Paracingulate Gyrus; Frontal Median Cortex
						[+2, +36, +22](17.2)			
			**pPerm = 0**	**pPerm = 0**	pPerm = 0.7902	[−2, +42, +18](16.3)			
						[+6, +50, −2](8.7)			
4	9	**FEF**	**corr = 0.5364**	corr = 0.5079	corr = 0.2081	1715 Voxels	1511 Voxels	900 Voxels	Juxtaposition Lobule Cortex; Paracingulate Cortex; Cingulate Gyrus anterior division; Precentral Gyrus; Middle Frontal Gyrus; (Area 6mr/preSMA/Area 3b/Area 4a/)
						[+2, +2, +62](30.1)	[+50, −2, +50](19.9)	[−48, −6, +50](18.1)	
			**pPerm = 0**	pPerm = 0.0012	pPerm = 0.3834	[+6, +12, +44](12.5)	[+56, +2, +38](14.4)	[−36, −16, +40](8.05)	
						[−8, +14, +38](8.81)			
4	76	DAN	corr = 0.4247	corr = 0.3866	corr = 0.2319	1330 Voxels	940 Voxels		Area 7A(SPL), Area 7PC(SPL), Area hIP3(IPS)—Lateral Occipital Cortex superior division; Superior Parietal Lobule
						[+22, −60, +64](17.5)	[−24, −62, +62](17.6)		
			pPerm = 0.0078	pPerm = 0.1017	pPerm = 0.2445	[+38, −48, +60](12.3)	[−36, −50, +60](11.2)		
5	109	pInsula	corr = 0.2994	corr = 0.344	corr = 0.3581	1119 Voxels	650 Voxels		Area Ig2, Area Id1, Area Ig1 ‐ Insular Cortex; Heschl's Gyrus; Planum Polare; Temporal Pole
						[+46, −8, −2](13.4)			
			pPerm = 0.2564	pPerm = 0.316	pPerm = 0.0641	[+42, −12, −4](11.9)	[−44, −10, −2](10.4)		
						[+40, −14, −2](11.9)			
						[+46, +8, −8](11.6)	[−38, −20, +4](9.05)		
5	8	**STG**	corr = 0.433	**corr = 0.5501**	corr = 0.2154	2023 Voxels	1615 Voxels		Area TE 3, Area TE 1.2 ‐ Planum Temporale; Superior Temporal Gyrus posterior division; Superior Temporal Gyrus anterior division;
						[−62, −16, +2](23.8)	[+60, −6, +0](20.4)		
			pPerm = 0.0029	**pPerm = 0.0001**	pPerm = 0.3573		[+66 − 24, +4](20)		
							[+66, −14, +0](17.9)		
5	32	PFcm(IPL)	corr = 0.279	corr = 0.2886	corr = 0.2461	1040 Voxels	257 Voxels		Area PFcm(IPL), Area OP1[SII], Area TE 1.0, Area PF(IPL), Area TE 3, Area TE 1.1, Area OP2[PIVC], Area Ig1 ‐ Planum Temporale; Heschl's Gyrus; Parietal Operculum Cortex
						[+50, −24, +10](20.5)	[−48, −28, +10](13.9)		
			pPerm = 0.2555	pPerm = 0.5194	pPerm = 0.3726	[+44, −30, +18](20.5)	[−36, −30, +16](13.2)		
						[+62, −28, +14](16)	[−38, −32, +14](13.1)		
5	31	**Operculum**	**corr = 0.5284**	corr = 0.5194	corr = 0.1636	1956 Voxels	1538 Voxels		Area OP4[PV], Area OP1[SII], Area OP3[VS], Area TE 1.0, Area 44, Area TE 3, Area OP2[PIVC], Area OP8, —Central Opercular Cortex; Planum Polare; Insular Cortex
						[+54, −8, +10](18.6)	[−54, −10, +10](19.6)		
			**pPerm = 0.0001**	pPerm = 0.002	pPerm = 0.6861	[+60, +2, +4](18)	[−34, +8, +8](9.97)		
						[+38, −8, +14](13)	[−40, −4, +12](9.65)		
						[+36, +8, +10](11.2)			
6	64	Pga(iPL)	corr = 0.3023	corr = 0.3139	corr = 0.1484	2056 Voxels	140 Voxels		Area PGa(IPL), Area PGp(IPL), Area hOc41a—Angular Gyrus; Middle Temporal Gyrus temporooccipital part; Lateral Occipital Cortex superior division; Lateral Occipital Cortex inferior division
						[+56, −54, +14](16.9)	[−56, −56, +10](7.42)		
			pPerm = 0.1447	pPerm = 0.2126	pPerm = 0.6853		[−48, −70, +16](7.19)		
6	124	Hippocampus	corr = 0.4392	corr = 0.4723	corr = 0.1966	642 Voxels	49 Voxels		Area FG3, CA1 (Hippocampus), DG (Hippocampus) Temporal Occipital Fusiform Cortex; Lingual Gyrus; Parahippocampal Gyrus posterior division; Temporal Fusiform Cortex posterior division;
						[+32, −40, −12](11.4)	[−28, −42, −12](7.66)		
			pPerm = 0.0138	pPerm = 0.0399	pPerm = 0.68	[+22, −42, −12](11.1)			
						[+28, −38, −14](11.1)			
6	25	**Precuneus**	corr = 0.4189	**corr = 0.635**	corr = 0.2628	920 Voxels	671 Voxels	29 Voxels	Precuneus Cortex; Supracalcarine Cortex; CA1 (Hippocampus), Subiculum, DG (Hippocampus)—Parahippocampal Gyrus posterior division; Lingual Gyrus
						[−16, −60, +18](30.4)	[+16, −56, +16](25.2)	[−28, −40, −12](10.4)	
			pPerm = 0.0054	**pPerm = 0**	pPerm = 0.1512				
6	82	**PGp(IPL)**	**corr = 0.4415**	corr = 0.4358	corr = 0.1221	705 Voxels	232 Voxels	200 Voxels	Area PGp(IPL), Area PGa(IPL), Area hIP5(IPS) Lateral Occipital Cortex superior division; Angular Gyrus; Precuneous Cortex; Supracalcarine Cortex; Cingulate Gyrus posterior division; Middle Frontal Gyrus; Superior Frontal Gyrus
						[+44, −64, +28](12.4)	[+10, −64, +24](9.46)	[+26, +26, +46](9.93)	
			**pPerm = 0.0009**	pPerm = 0.0154	pPerm = 0.9177		[+8, −56, +28](8.25)		
6	16	**DMN**	corr = 0.3872	**corr = 0.5627**	corr = 0.1938	2127 Voxels	101 Voxels	99 Voxels	Precuneus Cortex; Cingulate Gyrus, posterior division, Frontal Pole, Area PGa(IPL), Area PFm(IPL), Area PGp(IPL)—Lateral Occipital Cortex superior division; Angular Gyrus
						[+0, −58, +28](29.4)	[+0, +62, −8](10.3)	[−46, −66, +28](7.04)	
			pPerm = 0.0112	**pPerm = 0.0001**	pPerm = 0.438			[+46, −60, 22](6.7)	

These findings in the motor as well as the salience‐attention‐executive networks, taken together with the behavioral results, reflect adequate sensory stimulation and proper task performance as required prerequisites for further evaluation.

### 3.3 Visual Cortex Components Including V5/MT

The visual areas V1, V3, V4, and V5/MT showed a sustained positive BOLD response (relative to onset of stimulation) with a plateau until the end of the stimulation phase (Figure 3). During the post‐stimulation phase, the activity of areas V1, V3, and V4 rapidly returned to baseline for all three conditions in a very similar way. Only the V5/MT responses showed time differences for the return to baseline in correspondence to their different MAE duration: First the random response returned, followed by the translation response, and finally the rotation response, and this was also reflected in a highly significant PLS correlation (Figure 3, Table 1).

### 3.4 Core Vestibular Cortical Network Components

**FIGURE 5:**
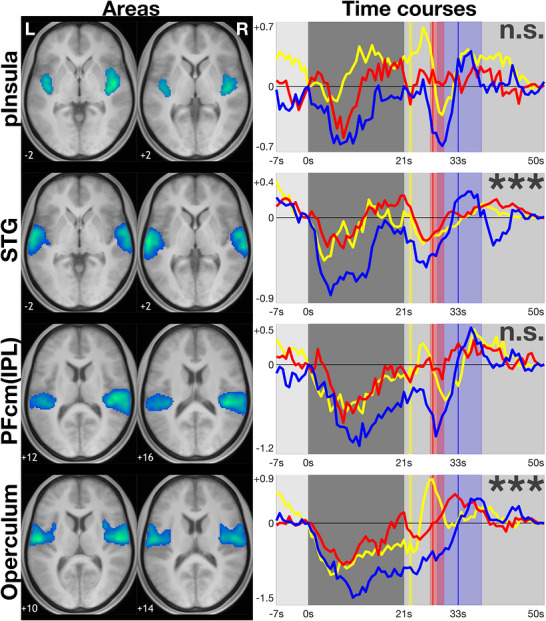
Temporal and spatial properties of the vestibular network components. All vestibular areas showed an immediate negative response with the beginning of the stimulation phase, especially for the rotation condition. The posterior insula (pInsula) and superior temporal gyrus (STG) showed transient two‐time negative responses (especially for rotation) at the beginning of the stimulation phase and at the beginning of the post‐stimulation phase, afterward returning to baseline, whereas the inferior parietal lobule (IPL) and the parietal operculum showed a sustained negative response over the whole stimulation phase continuing to the post‐stimulation phase before returning to baseline. The posterior insula showed the strongest negative response to rotational and almost none to random stimulation, while the other vestibular areas, although also most strongly modulated by rotation additionally, showed negative modulation even for random motion stimulation. Left column: overlays of activated areas; right column: responses relative to onset of visual motion stimulation. Time course illustration: Response curves color‐coded in blue for rotation, red for translation, and yellow for random. Stimulation phase indicated by dark gray (0 = Onset to 30TRs), and the post‐stimulation phase by light gray background (30–70 TRs). Translucent vertical color regions illustrate when 25% (left edge) and 75% (right edge) of the group perceived the end of MAE during a condition, respectively. The central vertical line indicates when 50% of participants perceived the end of MAE. *** highly significant PLS correlation with MAE duration (*p* < 0.0001); n.s. = not significant.

The known core vestibular cortical network areas, that is, the posterior insula/parietal operculum, the superior temporal gyrus (STG), and the inferior parietal lobule (IPL PFcm: posterior [magnocellular] supramarginal area; area supramarginalis columnata magnocellularis posterior) (Triarhou 2007), all showed negative responses during the stimulation phase for rotation stronger than for translation (Figure 5). For the random stimulation, the posterior insula showed the least amount of negative response compared to all other vestibular areas, while the other areas also showed a clear negative response for the random condition. Hence, the core vestibular area, posterior insula, is most responsive to coherent visual motion stimulation (movement‐relevant visual motion), while the other areas show responsiveness to visual motion per se. Notably, only the parietal operculum and the STG components showed a significant PLS correlation with MAE duration, but not the posterior insula and the PFcm(IPL) (Table 1). Notably, the operculum component correlated with MAE duration mainly during the stimulation phase and less so during the post‐stimulation phase, while all other components that showed correlation with MAE duration did so only during the post‐stimulation phase (see Table 1 for comparison). While the posterior insula and STG exhibited a transient (returning to baseline before the end of stimulation phase) negative response twice, at the beginning as well as at the end of stimulation (most notably for rotation), the IPL and parietal operculum showed a sustained negative response over the whole stimulation phase, returning to baseline only in the post‐stimulation phase (Figure 5). In summary, within the core vestibular network there is a fourfold distinction of BOLD responses in two dimensions: (1a) transient versus (1b) sustained and (2a) MAE duration correlated versus (2b) not correlated.

### 3.5 Further Downregulated Components

**FIGURE 6:**
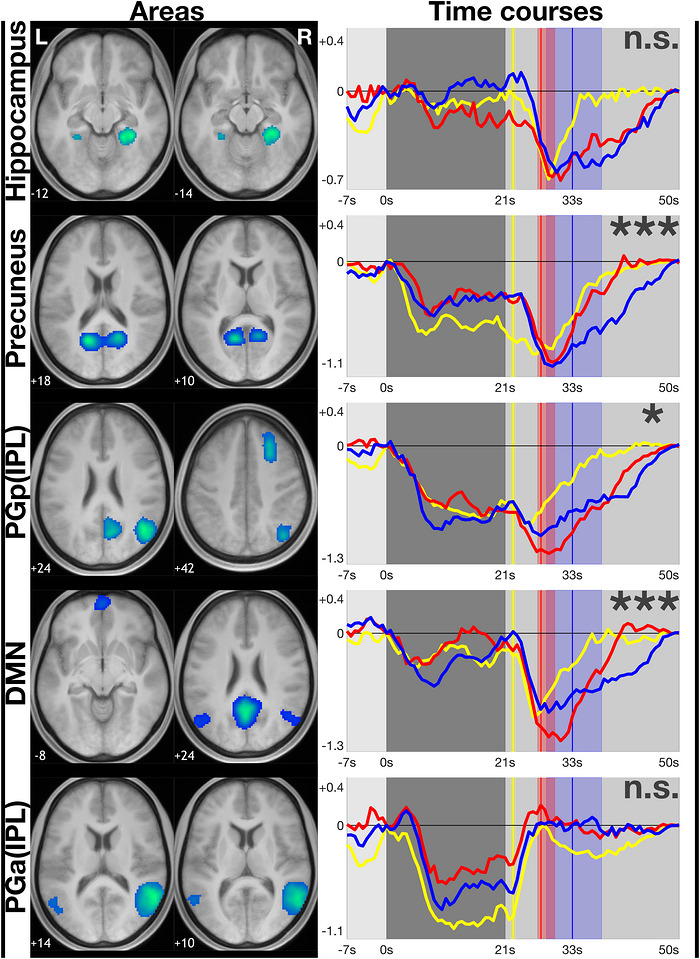
Temporal and spatial properties of other areas with negative responses. The response curves of the hippocampus, precuneus, and PGp (IPL) were all negative during the stimulation phase with similar amplitudes for all three conditions. The default mode network (DMN) modulated mostly during the post‐stimulation phase, less during the stimulation phase. For all these components, the translation and rotation conditions induced an even stronger negative signal at the beginning of the post‐stimulation phase and a later return to baseline compared to random. The PGa (IPL) exhibited a negative response during the stimulation phase, but no specific response during the post‐stimulation phase. Left column: overlays of activated areas; right column: responses relative to onset of visual motion stimulation. Time course illustration: Response curves color‐coded in blue for rotation, in red for translation, and yellow for random. Stimulation phase indicated by dark gray (0 = Onset to 30TRs), and the post‐stimulation phase by light gray background (30–70 TRs). Translucent vertical color regions illustrate, when 25% (left edge) and 75% (right edge) respectively of the group perceived the end of MAE during a condition. The central vertical line indicates when 50% of participants perceived the end of MAE. * weakly significant PLS correlation with MAE duration (p<=0.001); *** highly significant PLS correlation with MAE duration (*p* < 0.0001); n.s. = not significant.

Further components with a negative response were the hippocampus, other parts of the IPL (PGp and PGa; PG: angular area; a: anterior/rostral; p:posterior/caudal [Triarhou 2007]), the precuneus, and the DMN. These showed variation in time course types: the hippocampus showed negative modulation only during the post‐stimulation phase, while the PGa(IPL) only showed negative modulation during the stimulation phase. The others (DMN, PGp(IPL), and precuneus) showed moderate negative modulation during the stimulation phase and pronounced negative modulation during the post‐stimulation phase. Out of these the IPL (PGp), the precuneus, and the DMN showed a significant PLS correlation with MAE durations (Figure 6, Table 1).

**FIGURE 7:**
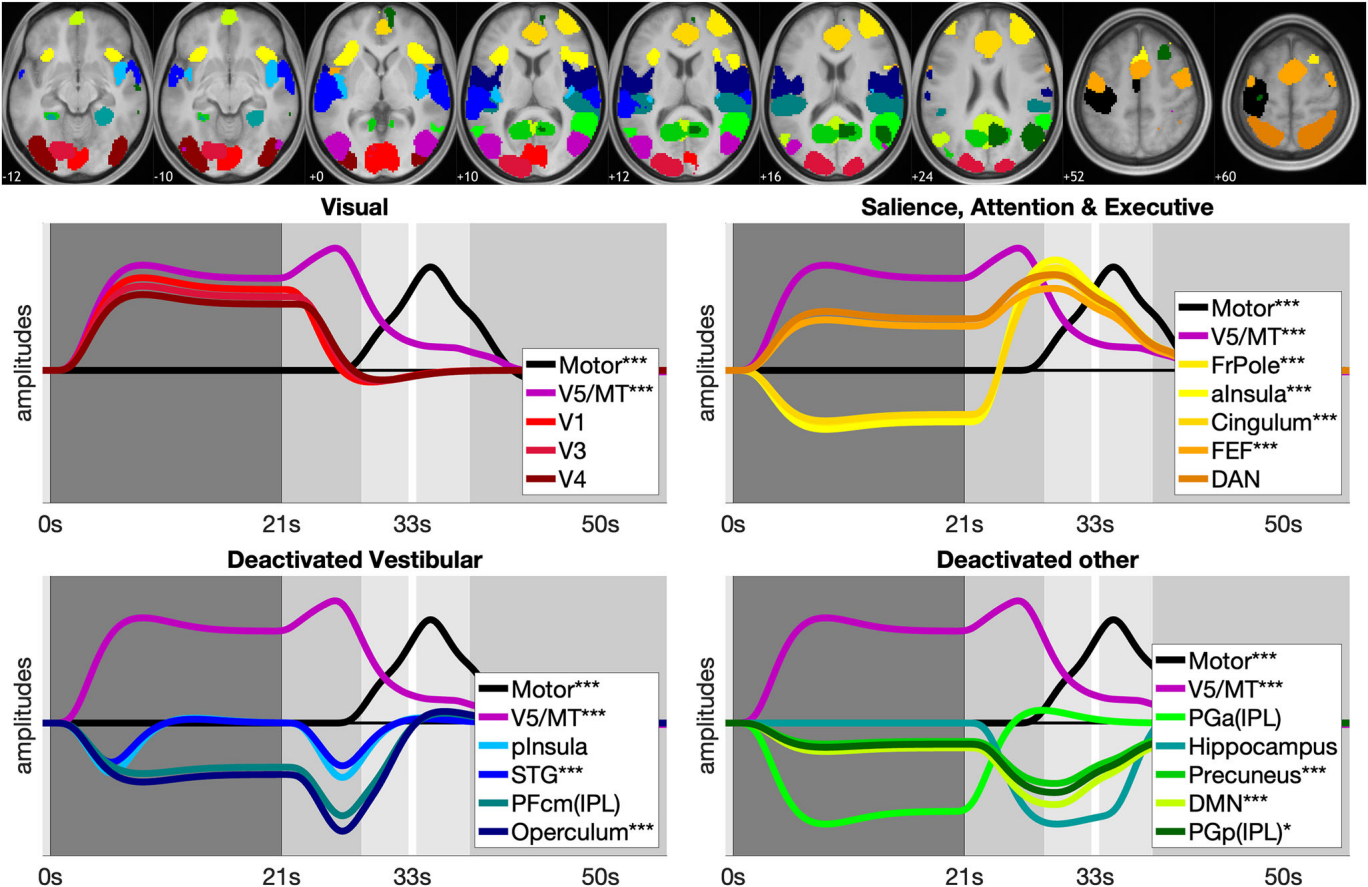
Schematic depiction of the main results for rotational stimulation for illustrative purposes: activated visual (red) and salience‐attention‐executive (yellow‐oranges) network areas, as well as deactivated vestibular (blue) and other network areas (cyan‐greenish). Top row: color coded anatomical overlay; below: stylized time courses of all relevant areas with corresponding colors. All time course plots contain the stylized responses in the left motor area (black) and V5/MT (magenta) for orientation. Vestibular and visual areas both already reacted during the stimulation phase. Vestibular areas were generally negative in response, while visual areas were all positive. Whereas vestibular areas showed diverse time courses (two‐timed downregulated at the beginning of stimulation and post‐stimulation phases versus sustained downregulated) and significant relation to MAE duration, visual areas all showed uniform time courses rapidly returning to baseline after stimulus stop, only V5/MT showed relation to MAE duration. Stimulation phase indicated by dark gray (0 = Onset‐30TRs), the post‐stimulation phase by light gray background (30–75 TRs), and the time window of MAE duration related responses in semitransparent white with the white vertical line indicating when 50% of participants indicated end of MAE akin to the real experimental data, that is, the window and line signifying the natural variability between subjects. *weakly significant (*p* < 0.001) **significant (*p* < 0.0005), and ***highly significant (*p* < 0.0001) PLS correlation with MAE duration.

In summary, the following components showed significant relationships with MAE durations using PLS correlation of ERAs (Table 1): the motor component (left motor cortex, SMA, and right cerebellum), visual network area V5/MT, salience‐attention‐executive components (frontal pole, anterior Insula, anterior cingulum, and FEF), precuneus, IPL (PGp), and DMN. For the vestibular area components, a significant correlation was found for the parietal operculum and STG components, but not for the posterior insula and the IPL (PFcm). No significant PLS correlation was found for visual areas V1, V3, and V4; DAN; hippocampus; and IPL (PGa) component. For illustrative purposes, the response curves of all relevant areas are depicted schematically in Figure 7.

## 4 Discussion

The current fMRI study evaluated the so far largely unknown spatiotemporal properties of visual–vestibular interaction by use of fast imaging and multivariate model‐free analysis of BOLD signals in healthy participants. Our approach enabled us to apply neuroimaging assessment of visual–vestibular interaction beyond simple block‐average activity during the stimulation phase, expanding the dynamical time course assessment into the post‐stimulation phase and combining it with behavioral measures of illusory (vestibular) motion strength, that is, MAE duration.

In the following, we first discuss key results within different brain networks in light of multimodal imaging, behavioral, and non‐human primate studies. We then address the overarching conceptual consequences for our understanding of multisensory integration and visual–vestibular calibration.

### 4.1 SpatioTemporal Properties of Visual Network Areas

All visual motion stimuli induced persistent bilateral symmetric signal increases relative to onset in the primary and secondary visual cortex areas V1–V4 that uniformly decreased to baseline shortly after stimulus stop. As expected, the decreasing complexity of the visual stimuli (random > rotation > translation) caused decreasing BOLD response amplitudes in these visual areas (Figure 3). Furthermore, there was no PLS correlation with MAE duration for any of these areas. In summary, the coherent or incoherent properties of the visual motion stimuli did not seem to play a relevant role in the timely responses across the different visual areas V1–V4.

Especially, the motion‐sensitive visual area V5/MT at the occipitotemporal junction exhibited a deviating signal response in the post‐stimulation phase with a delayed return to baseline and highly significant PLS correlation. The return time was longest for rotation, medium for translation, and shortest for random stimulation (Figure 3). Indeed, principal involvement of V5/MT was not unexpected, since it was already found by previous imaging studies in humans applying visual motion stimuli that induce a sensation of self‐motion (e.g., MAE, circular or linear vection) (Bense et al. 2006; Culham et al. 1999; Deutschländer et al. 2004; Oh et al. 2018; Rühl et al. 2018; Stephan et al. 2005; Tootell et al. 1995). On the other hand, multimodal imaging studies applying different types of vestibular stimuli (caloric vestibular stimulation, galvanic vestibular stimulation, or VEMPs) regularly reported V5/MT involvement, too. This multisensory processing is in line with non‐human primate electrophysiology data demonstrating that MSTd—the homologue of V5/MT in animals—in fact receives multisensory, that is, visual as well as vestibular, inputs (Liu et al. 2023). The unique graded responses in V5/MT in our study highlight its special role as a or the leading *intermediate* visual–vestibular area.

### 4.2 SpatioTemporal Properties of Core Vestibular Network Areas

The known core vestibular network areas in the temporoparietal cortex (such as the posterior insula, parietal operculum, retroinsular cortex, STG, and IPL) (Lopez et al. 2012; Neumann et al. 2023; Zu Eulenburg et al. 2012) all exhibited bilateral signal *decreases* during the stimulation phase, particularly during rotation (Figure 5). This is compatible with the general concept of reciprocal inhibitory intersensory interaction, here with predominant visual input inducing visual network up‐regulation and vestibular network downregulation (Brandt et al. 1998; Dieterich and Brandt 2024, 2020), as well as with the concept of inter‐system recalibration (Harris et al. 1981).

Notably, for the vestibular areas, differentiated spatiotemporal response features were uncovered in the current study. Although PFcm(IPL) and parietal operculum showed relatively sustained decreases over the whole stimulation phase, the posterior insula, stronger than the STG, exhibited two‐timed transient decreases at the beginning of the stimulation, going back to baseline in between and decreasing again at the beginning of the post‐stimulation phase (Figure 5). Furthermore, significant PLS correlation with MAE duration was only evident for the STG and the parietal operculum. These signal differences might reflect subspecializations within the vestibular network for certain stimulus features.

Interestingly, just recently non‐human primate studies have shown a multitude of tuning properties for the vestibular areas: (1) a weighting of activity with stimulus acceleration or velocity, (2) a varying degree of vestibular and visual input (mono‐ vs. multisensory), and (3) a variation of respective reference frames (head, body, eye, and world). Some vestibular cortical network areas were demonstrated to be largely vestibular‐dominated (including PIVC as the homologue of the “primary” vestibular area, 3a, 2v), while others are considered equally multisensory (e.g., vestibular and visual, e.g., MSTd, VPS, VIP, 7a, FEF, PCC) (Liu et al. 2023). The two‐timed transient decrease within the posterior insula and STG in our human study is notably consistent with these animal data and might reflect a closer relation of these areas to acceleration properties of the stimulus, which is present at the beginning of the motion stimulus and again at its stop. The other areas at the parietal operculum and PFcm(IPS) might be more closely related to velocity properties indicated by their sustained signal decrease until stimulus stops. One can speculate that those areas with positive MAE correlation might play a role in maintaining the illusory vestibular self‐motion experience, which so far has been ascribed to V5/MT only, and thus indicate inter‐system offset recalibration as required by behavioral studies.

Furthermore, out of all vestibular areas, the posterior insula, as the “primary” multisensory vestibular core area, showed the least negative response to random visual motion stimulation and thereby seems to be particularly tuned to coherent visual motion, usually closely related to real body movement (Harris et al. 1981). Fittingly, in non‐human primates, PIVC was shown to be vestibular dominated, while other vestibular network areas were considered more equally multisensory (Liu et al. 2023).

### 4.3 Areas Outside the Core Vestibular Network With Negative Signal Modulations

There were further areas outside the known core vestibular network (especially the DMN, precuneus, and PGp(IPL)) showing a threefold combination of negative signal modulations, positive correlation with MAE duration (vestibular perception), and peak modulations during the post‐stimulation phase, together suggesting a potential intermediate role at the junction between vestibular and visual processing, beside MT/V5 (Figure 6).

The precuneus is a brain region involved in a variety of complex functions, for example, recollection and memory, integration of information related to perception of the environment, cue reactivity, mental imagery strategies, episodic memory retrieval, and affective responses (Cavanna and Trimble 2006). In the context of vestibular research by imaging or psychophysics, it has been especially associated with dynamic reweighting of conflicting sensory inputs (e.g., inhibiting visual dominance during vestibular activation) to prioritize valid motion cues and facilitate error correction in balance control (Becker‐Bense et al. 2020; Becker‐Bense et al. 2012; Ferrè et al. 2015; McAssey et al. 2022). Furthermore, reduced precuneus activity correlated to dizziness severity in vestibular lesioned patients (Fu et al. 2022; K. Li et al. 2020; Lin et al. 2023). We interpreted the precuneus modulations (deactivation in the stimulation phase, followed by stronger deactivation in the post‐stimulation phase and MAE duration correlation) as a sign of inter‐system recalibration affecting higher order visual–vestibular functions related to error detection, mental imagery, and even memory retrieval.

Parts of the IPL have been associated with visuospatial processing and visuomotor integration (Andersen 1987). They have been shown to modulate responses based on head/eye position during motion and resolving sensory conflicts during self‐motion (Della‐Justina et al. 2015; Van Ombergen et al. 2017). Lesions in the IPL have been associated with visual disorientation and impaired spatial navigation (Andersen 1987; Della‐Justina et al. 2015; Van Ombergen et al. 2017). We interpret the deactivations in the IPL areas as a sign that inter‐system offset recalibration affects higher order visual–vestibular functions, visuospatial function, navigation, and reference frame transformations.

The DMN has been associated with visual motion processing, internal self‐referential monitoring, and visuo‐spatial and temporal prediction processing (Andrews‐Hanna 2012; Carvalho et al. 2016; Dayan et al. 2016; J. Li et al. 2019; Van Buuren et al. 2010) as well as most notably with MAE (Rühl et al. 2018). In addition, it should be noted that our task requires continuous attention, which could also explain DMN modulation.

Remarkably, solely the hippocampus reacted with a negative response only during the post‐stimulation phase and rather with no response during the stimulation phase. This special signal behavior of the hippocampus might be correlated to its known key role in velocity storage mechanisms and vestibular memory (Brandt et al. 2005; Kremmyda et al. 2016; review by Smith 2022), which might be involved in storing the inter‐system offset. One can speculate that the inter‐system offset and thus MAE is leading the hippocampus to prepare to store memory, but after MAE is resolved there is no need for storage, and therefore the hippocampus reverts to forgetting this memory.

When we now combine the above insights into more general principles, the following can be concluded from this study.

### 4.4 Concluding Insights Into Visual–Vestibular Interaction Processing

It is obvious that different sensory networks, especially the visual and the vestibular ones, are simultaneously involved in motion processing with concurrent self‐motion perception. Up to now the mode of visual–vestibular interaction was seen as a categorical “on‐off” reciprocal interplay between both systems to resolve intersensory conflicts (Becker‐Bense et al. 2012; Brandt et al. 1998; Deutschländer et al. 2002).

It was an interesting and initially surprising new finding that the reciprocal visual–vestibular interaction pattern was engaged immediately with stimulation and not only when or after a strong intersensory conflict, and a vestibular percept of vection is built up. Even our random visual motion stimulus not inducing illusory self‐motion perception led to negative signals in the vestibular cortical network. However, earlier PET tracer accumulation or early slow fMRI studies relied on average signal changes during stimulation and thus were not able to resolve signal variations over time in such detail (Becker‐Bense et al. 2012; Brandt et al. 1998; Deutschländer et al. 2002).

In our study, the core vestibular areas could be divided into those showing a two‐time transient (reacting at the beginning of stimulation and post‐stimulation phase: posterior insula and STG) and those showing a sustained negative response: PFcm(IPL) and parietal operculum, respectively. Furthermore, only one area of each category was correlated with the illusory self‐motion percept, suggesting a specialization within the vestibular network for motion signal features and their role in inter‐system interaction.

These plentiful spatiotemporal signal properties in humans are well in line with non‐human primate data reporting activity weighting in relation to certain stimulus features and differentiation of largely vestibular‐dominated versus more equally multisensory composed areas (Liu et al. 2023). This is an essential difference of the nature of the vestibular compared to the visual system, since the latter is monosensory and primarily processes visual input (Rolls 2024). Although it is not possible to easily transfer non‐human primate data to humans, our findings match the overall concept of different weightings and subspecialization within the vestibular cortical network (e.g., for acceleration or velocity properties).

It is noteworthy that, beside V5/MT, further areas outside the visual cortex with intermediate signaling were identified (precuneus, DMN, parts of IPL, and also parietal operculum and STG), indicating their involvement in the extended visual–vestibular interaction network, most likely related to higher cortical vestibular functions.

Thus, the current study showed that visual–vestibular interaction is not a simple upregulation of all areas of one system and simultaneous downregulation of the other. Through being able to connect dynamic signaling over time to motion perception, we revealed a continuous dynamic weighted interplay of the systems calibrating information between each other. To our best knowledge, this is the first study demonstrating a correlate of the behaviorally conceptualized inter‐system offset recalibration within the human brain (Bai et al. 2019, 2020; Harris et al. 1981; Mather et al. 2008; Mather and Harris 1998; Van De Grind et al. 2004). This is in line with the fact that the vestibular system has a storage mechanism (Laurens and Angelaki 2011; Smith 2022), which the visual system does not (Van De Grind et al. 2004), thus explaining how MAE can be stored as a function of inter‐system offset recalibration. This function appears to be especially supported by the vestibular cortical network areas that show stimulation‐ and percept‐related variances in negative signaling, whereas within the visual system, rather uniform positive response curves dominated.

Our study gives deeper insights into the overall understanding of how moving—which requires distinguishing self‐motion from object‐motion—might be achieved even in the real world. Most notably, that visual–vestibular interaction persists all the time, even if one of these systems indicates stationarity, and inter‐system weighting is constantly being gradually adjusted.

Finally, we want to note some possible limitations. Our study, as in human imaging studies in general, requires examination while lying still and use of an artificial stimulation paradigm inducing a vestibular sensation in the absence of real body movement. Thus, the results cannot easily be transferred to natural conditions during real motion, when visual, vestibular, somatosensory, and motor systems receive simultaneous inputs and work dynamically together. Hopefully, new technical examination possibilities will allow investigation of visual–vestibular interaction under real‐life conditions in the future. Furthermore, we used the duration of subjective self‐motion perception, prone to errors of judgement and general noise from reaction times possibly affecting correlation analyses. To address this potential limitation, we controlled the eye fixation of the participants and measured button press times in all conditions to ensure vigilance and proper task performance. Fortunately, there were quite consistent results within and between participants and in relation to the different motion stimuli, indicating adequate estimates of illusory motion perception. Our results might not be generalizable to all other kinds of MAE. It is possible that experiments using so‐called dynamic MAE (tested with random noise, flickering dots, or random dot motion) and MAE induced by local motion might reveal different aspects, as it was previously shown for behavioral results (Hiris and Blake 1992). Our study included 22 healthy controls, which is close to the meanwhile median sample size of 24 subjects for comparable neuroimaging studies (Szucs and Ioannidis 2020). However, from our point of view, the logical and consistent results under graded visual stimulation conditions in the two different phases and in correlation to the perceptual parameters as well as the parallels between human and animal data speak for the validity of our results.

## Author Contributions

**Conceptualization**: Sandra Becker‐Bense, Marianne Dieterich, Thomas Brandt, and Oliver Pogarell. **Recruiting of participants**: Franziska Reichl, Lena Fabritius, and Maximilian Maywald. **MRI measurements**: Franziska Reichl, Lena Fabritius, and Rainer Boegle. **Data curation**: Rainer Boegle and Franziska Reichl. **Formal analysis**: Rainer Boegle. **Funding acquisition**: Marianne Dieterich. Investigation: Rainer Boegle, Franziska Reichl, and Lena Fabritius. **Methodology and analyses**: Rainer Boegle and Sandra Becker‐Bense. **Project administration**: Sandra Becker‐Bense and Oliver Pogarell. **Resources**: Sandra Becker‐Bense and Marianne Dieterich. **Software**: Rainer Boegle. **Supervision**: Sandra Becker‐Bense, Marianne Dieterich, Thomas Brandt, and Oliver Pogarell. **Validation**: Rainer Boegle. **Visualization**: Rainer Boegle and Franziska Reichl. **Writing – original draft**: Rainer Boegle and Sandra Becker‐Bense. **Writing – review and editing**: Marianne Dieterich and Thomas Brandt.

## Funding

This work was supported by the Graduate School of Systemic Neurosciences (GSN) which was funded by the German Research Foundation (DFG) to R.B. ands M.D., the German Federal Ministry of Education and Research (BMBF) in connection with the foundation of the German Center for Vertigo and Balance Disorders (DSGZ; grant number 01 EO 0901) to T.B. and M.D. M.D. was supported by the Deutsche Stiftung Neurologie (DSN; project 80766113) and by the Deutsche Forschungsgemeinschaft (DFG, German Research Foundation) under Germany's Excellence Strategy (Munich Cluster for Systems Neurology: EXC 2145 SyNergy). R.B. was supported by the Deutsche Stiftung Neurolsogie (DSN; project 80721019).

## Ethics Statement

The study was conducted according to regulations of the Helsinki Declaration (revised 2024) and was approved by the local Ethics Committee of the Ludwig‐Maximilians‐Universität München, Germany (23‐0660). All participants gave their informed written consent.

## Conflicts of Interest

All authors declare no conflicts of interest.

## Supporting information




**Supplementary Figure**: brb371621‐sup‐0001‐FigureS1.docx

## Data Availability

Data will be made available on request.
