# Supplementary material for: Expanded Spatiotemporal Concept of Cortical Visual–Vestibular Interaction in Humans: A fMRI Study on Visually Induced Motion Perception
Source: Brain Behav. 2026 Jul 24;16(7):e71621. doi: 10.1002/brb3.71621 (PMC13396996; doi:10.1002/brb3.71621)
Supplement: Supplementary file 1 — Supplementary Figure: brb371621‐sup‐0001‐FigureS1.docx [file BRB3-16-e71621-s001.docx]

**Full title:**

**Expanded spatio-temporal concept of visual-vestibular interaction in humans: a fMRI study on visually-induced motion perception**

**Authors:**

Rainer Boegle^a,e^, Franziska Reichl^a^, Lena Fabritius^a,b^, Maximilian Maywald^c^, Oliver Pogarell^c^, Thomas Brandt^a^, Marianne Dieterich^a,b,d,e^, Sandra Becker-Bense^a,b^

**Affiliations:**

^a^German Center for Vertigo and Balance Disorders, University Hospital, LMU Munich, Germany

^b^Department of Neurology, University Hospital, LMU Munich, Germany

^c^Department of Psychiatry and Psychotherapy, University Hospital, LMU Munich, Germany

^d^Munich Cluster of Systems Neurology (SyNergy), Munich, Germany

^e^Graduate School of Systemic Neuroscience, LMU Munich, Germany

**Running title (max 40 characters):**

visual-vestibular interaction

**Correspondence to:**

Rainer Boegle

German Center for Vertigo and Balance Disorders (DSGZ)

University Hospital LMU Munich

Marchioninistr. 15

81377 München

Germany

Phone: (++49) 89 4400 77825

Fax: (++49) 89 4400 74801

E-mail: [rainer.boegle@med.uni-muenchen.de](mailto:rainer.boegle@med.uni-muenchen.de)

**Supplementary Material**


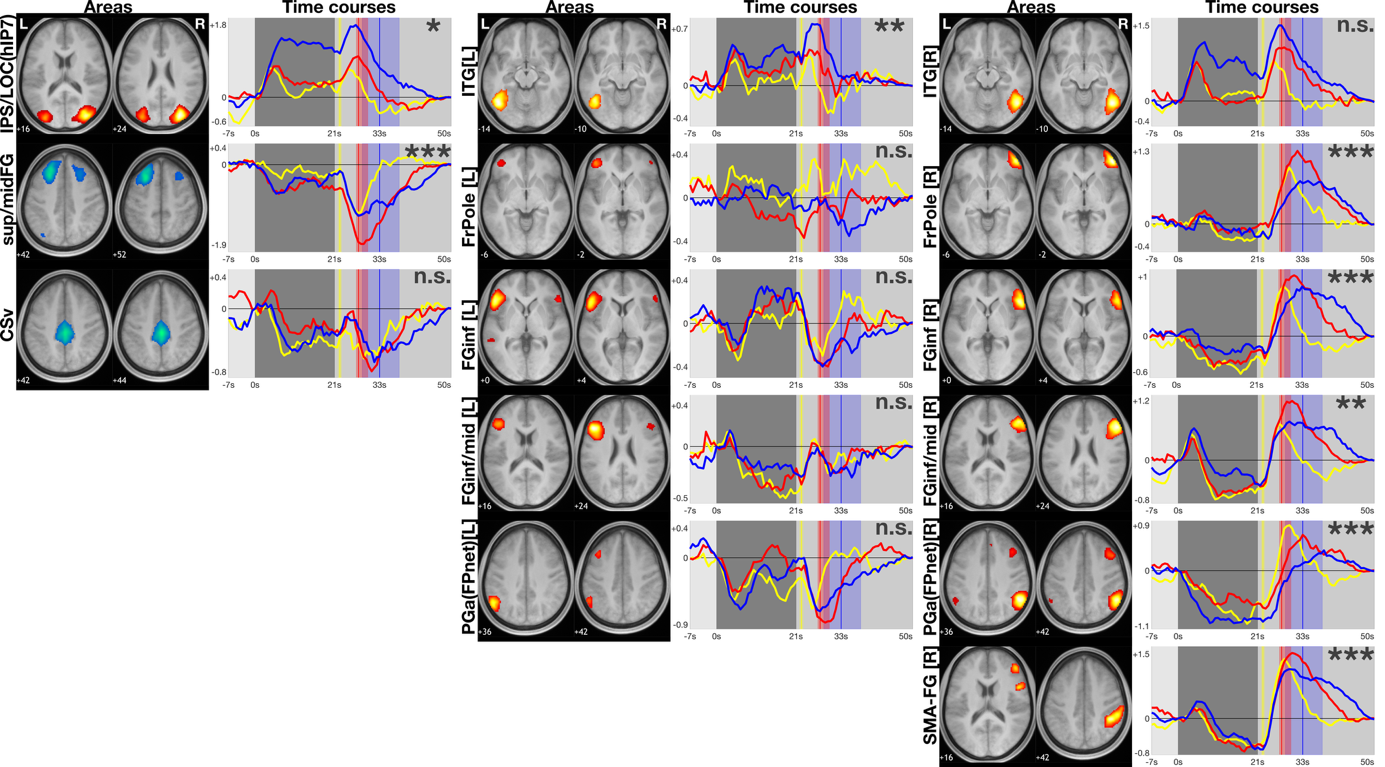


Supplemental Figure 1:

Overlays and temporal responses of areas that also fit into the salience-attention-executive category similar to those in Figure 4.

Bilaterally symmetric components are shown on the left of the figure, comprising inferior parietal sulcus lateral occipital cortex (IPS/LOC(hIP7)) and superior/middle frontal gyrus (sup/mid FG). The other components split into left- and right-sided parts, with the left-sided components shown in the middle and the right-sided components shown on the right of the figure. For the right-sided component comprising the network of right supramarginal gyrus to right frontal gyrus (SMA-FG) there was no counterpart in the opposite hemisphere among the 150 components, therefore there is no partner shown. Components with left-sided and right-sided partners were, inferior temporal gyrus (ITG), frontal pole (FrPole), inferior frontal gyrus (FGinf), inferior-to-middle frontal gyrus (FGinf/mid) and fronto-parietal net centered around the area PGa(FPnet). For the left-sided components only the responses of the ITG was significantly related to MAE duration, while all other left-sides components were not significant. For the right-sided components almost all components had time courses significantly related to MAE duration, except the ITG component. All bilateral components had time courses significantly related to MAE duration. Therefore, there was a clear difference between left-sided components, which had no significant correlations with MAE durations (except left-ITG) and right-sided components which were all significantly correlated with MAE durations (except right-ITG) and both bilateral components which were also significantly correlated with MAE durations.

Left column: overlays of activated areas; right column: responses relative to onset of visual motion stimulation. Time course illustration: Response curves color-coded in blue for rotation, in red for translation, and yellow for random. Stimulation phase indicated by dark gray (0=Onset to 30TRs), and the post-stimulation phase by light gray background (30 to 70 TRs). Translucent vertical color regions illustrate, when 25% (left edge) resp. 75% (right edge) of the group perceived the end of MAE during a condition. The central vertical line indicates when 50% of participants perceive the end of MAE. Abbreviation: *** = highly significant PLS correlation with MAE duration; n.s. = not significant
